# Likelihood Inference for Factor Copula Models with Asymmetric Tail Dependence

**DOI:** 10.3390/e26070610

**Published:** 2024-07-19

**Authors:** Harry Joe, Xiaoting Li

**Affiliations:** Department of Statistics, University of British Columbia, Vancouver, BC V6T 1Z4, Canada; xiaoting.li@stat.ubc.ca

**Keywords:** copula, latent variable, likelihood, prior, Bayesian computing, numerical optimization

## Abstract

For multivariate non-Gaussian involving copulas, likelihood inference is dominated by the data in the middle, and fitted models might not be very good for joint tail inference, such as assessing the strength of tail dependence. When preliminary data and likelihood analysis suggest asymmetric tail dependence, a method is proposed to improve extreme value inferences based on the joint lower and upper tails. A prior that uses previous information on tail dependence can be used in combination with the likelihood. With the combination of the prior and the likelihood (which in practice has some degree of misspecification) to obtain a tilted log-likelihood, inferences with suitably transformed parameters can be based on Bayesian computing methods or with numerical optimization of the tilted log-likelihood to obtain the posterior mode and Hessian at this mode.

## 1. Introduction

Dependence models with multivariate copulas have had many applications in the past two decades to handle non-Gaussian dependence; in particular, for applications such as risk analysis where variables can have more dependence in the joint tails than with Gaussian dependence with the same strength of central dependence.

When pairwise scatterplots of variables suggest lower and upper tail dependence, possibly asymmetric in the strength in the joint lower tails (extreme of lower quadrant) versus the strength in the joint upper tails (extreme of upper quadrant), several different parametric copula families with tail dependence are among the best based on information criteria such as the Akaike information criterion (AIC). However model-based bivariate lower and upper tail dependence measures can be quite different for these different parametric copulas, and the comparisons of lower and upper tail dependence measures might not match the visual comparisons on the pairwise scatterplots. This is because likelihood methods are influenced a lot by data in the middle (rather than extremes), and all simple parametric models have some degree of misspecification.

For univariate distributions, it is well known that inferences involving large quantiles should not be based on a fitted parametric distribution because extrapolation is not reliable when the data values in the middle have the most influence in the parameter estimates. There are two approaches for univariate inferences involving extremes: (a) from univariate extreme value theory with the assumption of a well-behaved tail density, the peaks-over-threshold method based on generalized Pareto distribution [1] can be used, or (b) splicing models [2] can be used with different flexible densities for the body and tail, if inferences are also needed for non-extremes. For the joint tail region, there is a multivariate Pareto approach such as that in [3], but there is no convenient way to combine with a density for the body.

The goal in this article is to propose a method that incorporates “prior” information on the relations of bivariate lower/upper tail dependence pairs, thereby placing more weight on joint extreme observations when estimating the dependence parameters of the multivariate copula; the splicing of densities for the body and the joint tails is avoided. This approach should lead to parameter estimates of copula dependence parameters with more reliable inference for tail dependence and other tail-based quantities.

How different parametric copula models lead to quite different tail inferences is illustrated with some financial returns data over a few consecutive years. Consider the financial returns for different market indexes or stocks in the same sector of a market; for dependence analysis, commonly, a copula-GARCH model (see [4]) is applied to GARCH-filtered returns. Pairwise normal scores plots after rank transform to N(0,1) show tail dependence with the clouds of points being sharper than the elliptical shape in the extreme lower and upper quadrants. Often, there appears to be a stronger dependence in the joint lower tail than in the joint upper tail.

When different flexible parametric multivariate copula families, such as vine and factor copula models, are fit to multivariate GARCH-filtered returns, the best-fitting models based on AIC imply lower and upper tail dependence for any pair of returns. This is based on the results of [5,6], which imply that if bivariate copulas, for pairs of variables in the first tree of the vine or with a variable linked to a latent variable, have lower and upper tail dependence, then the bivariate copulas of all pairs of variables have lower and upper tail dependence. Note that factor copulas are vine copulas that include latent variables.

However, if model-based tail dependence parameters are computed based on the best few fitted models, they can be quite different among the models and sometimes may not match what is seen in the normal score plots. For example, (a) sometimes, a model-based lower tail dependence parameter may be closer to 0 than expected based on the plot, or (b) the model-based lower tail dependence parameter may be smaller than the model-based upper tail dependence parameter, in contrast to the the visual inspection of the plot.

With the non-parametric method for empirical tail dependence measures in [7], it is possible to compare empirical and model-based lower and upper tail dependence to show quantitatively that model-based measures might not be reliable for all bivariate margins. This is because the fit of parametric multivariate models based on likelihood tends to be dominated by the data in the middle of the distribution. Inference concerning the middle (e.g., medians and non-extreme marginal orthant probabilities) can be reliable but not necessarily inference concerning the extremes (e.g., extreme marginal orthant probabilities or multivariate quantiles of the form defined in [8]).

This article shows the use of a tilted likelihood to estimate parameters of the 1-factor copula so that inferences in the joint tails are improved.The 1-factor copula for *d* variables has a vector parameter $\vartheta_{j}$ for the bivariate linking copula of the *j*th variable and the latent variable (the latter explains the joint dependence of the observed variables). The tilting depends on the nature of the variables. For *d* GARCH-filtered stock returns for stocks in the same sector, the dependence parameters $\left\{\vartheta_{j}:1\leq j\leq d\right\}$ can be considered a sample from a super-population so that it is reasonable to assume a common prior distribution for the $\vartheta_{j}$. The tilted log-likelihood is based on the sum of the 1-factor copula log-likelihood and the logarithm of this prior density that is based on tail dependence summaries from some “previous data”.

There is a numerical data example in Section 2 for preliminaries to show explicitly why likelihood inference can be inadequate for tail inference; tail dependence parameters are defined, and examples of normal score plots are given in this section. Section 3 and Section 4 contain the theory and numerical methods to develop a “prior” to help with tail inference for the 1-factor copula model with asymmetric tail-dependent copulas linking to the latent variable. Section 5 illustrates the theory for a data example with GARCH-filtered stock returns from stocks in a S&P sector to show improved tail inference. Section 6 has some simulation results to compare with the data example. Section 7 concludes with a discussion on the generality of the approach proposed for the 1-factor model; the basis is a “super-population” assumption for some bivariate margins with lower and upper tail dependence. The background results for tail dependence, copulas, and factor models are given in Appendix A.

## 2. Numerical Data Example to Illustrate Discrepancy for Tail Inference

In this section, a numerical low-dimensional data example is used to clarify what is meant by the possible poor joint tail inference following maximum likelihood.

Definitions of bivariate tail dependence and the copula as summaries of dependence are presented to explain concepts of dependence in joint tails.

Let $F_{1:d}$ be an absolutely continuous *d*-variate distribution with univariate margins $F_{1},\ldots ,F_{d}$ and copula $C_{1:d}$ such that $F_{1:d}=C_{1:d}\left(F_{1},\ldots ,F_{d}\right)$. Let $Y=\left(Y_{1},\ldots ,Y_{d}\right)∼F_{1:d}$.

For the bivariate margin $F_{jk}=C_{jk}\left(F_{j},F_{k}\right)$ with j≠k, the probabilistic version of the lower and upper tail dependence parameters is:

$$\begin{array}{ccc} \lambda_{jk,L} & = & \lim_{u\to 0^{+}}\mathrm{Pr}\left(Y_{j}\leq F_{j}^{-1}\left(u\right)|Y_{k}\leq F_{k}^{-1}\left(u\right)\right)\\ & = & \lim_{u\to 0^{+}}u^{-1}\mathrm{Pr}\left(Y_{j}\leq F_{j}^{-1}\left(u\right),Y_{k}\leq F_{k}^{-1}\left(u\right)\right)=\lim_{u\to 0^{+}}C_{jk}\left(u,u\right)/u,\\ \lambda_{jk,U} & = & \lim_{u\to 0^{+}}\mathrm{Pr}\left(Y_{j}\geq F_{j}^{-1}\left(1-u\right)|Y_{k}\geq F_{k}^{-1}\left(1-u\right)\right)\\ & = & \lim_{u\to 0^{+}}u^{-1}\mathrm{Pr}\left(Y_{j}\geq F_{j}^{-1}\left(1-u\right),Y_{k}\geq F_{k}^{-1}\left(1-u\right)\right)\\ & = & \lim_{u\to 0^{+}}{\bar{C}}_{jk}\left(1-u,1-u\right)/u, \end{array}$$

where ${\bar{C}}_{jk}\left(u_{j},u_{k}\right)=1-u_{j}-u_{k}+C_{jk}\left(u_{j},u_{k}\right)$ for $0\leq u_{j},u_{k}\leq 1$.

Consider a random sample from $F_{1:d}$ with $y_{i}=\left(y_{i1},\ldots ,y_{id}\right)$ for i=1,…,n. Because $\lambda_{jk,L}$ and $\lambda_{jk,U}$ are limiting quantities (as $u\to 0^{+}$), there are no direct empirical (data) versions. For the numerical examples in this section and later sections, the sample version comes from a limit of tail-weighted dependence measures.

A general reference for concepts (in the above and in later sections) with copulas and dependence is [9], and the estimator of tail dependence from a limit of tail-weighted dependence measures is given in [7]. For the probabilistic version, the tail-weighted dependence measures are indexed by a parameter α>1 and the limit, as α→∞ is the tail dependence parameter. After computing the empirical tail-weighted dependence measure for a grid of α values, typically in the interval [10,20], a regression model is fit for the empirical measure versus a power of $\alpha^{-1}$, and then the tail dependence parameter is estimated as the extrapolation with $\alpha^{-1}\to 0$.

The data example involves GARCH-filtered stock returns with all stocks in the same sector. Appendix A.4 has some background for GARCH time series and copula-GARCH models.

**Figure 1:**
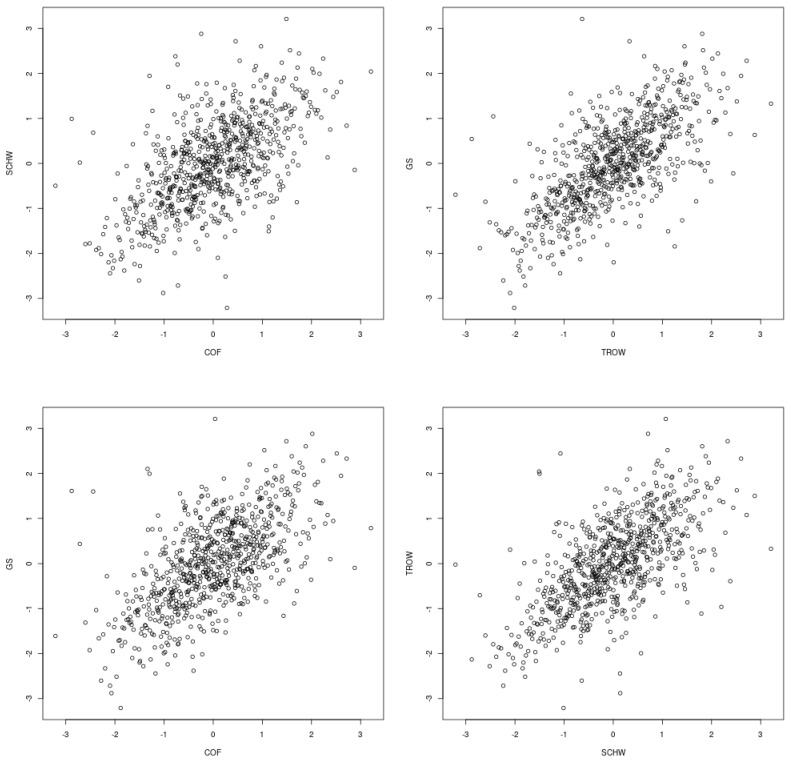
Normal score plots for some pairs of GARCH-filtered stock returns. Lower and upper semi-correlations, as used in Section 2.4 of [9], show more dependence in the lower quadrant than in the upper quadrant and suggest asymmetric tail dependence.

The S&P 500 data set of GARCH-filtered stock returns (January 2013 to December 2015, good economic conditions) used for illustration is analyzed in [10]. The sample consists of n=754 days. For the finance sector, some initial descriptive statistics analyses based on 10 stocks were chosen from 64; the ticker symbols of the 10 stocks are COF, RJF, SCHW, FRC, GL, FD, TROW, GS, BLK, and ICE. Normal score plots of GARCH-filtered returns for a few pairs amongst these 10 stocks are given in Figure 1 (see Appendix A.3 for the mathematical definition of the transform). They show tail dependence, with the clouds of the points being sharper than the elliptical shape and having a stronger correlation in the lower quadrant than in the upper quadrant.

These few stocks are used to demonstrate (in small tables) a typical situation of differences in empirical and model-based tail quantities. To check that a 1-factor dependence structure is reasonable, the non-parametric transform to normal scores is applied to GARCH-filtered returns, and factor analysis (see [11]) is applied to the resulting correlation matrix. The loadings are, respectively, 0.741, 0.802, 0.838, 0.475, 0.688, 0.821, 0.665, 0.609, 0.690, 0.830. The average absolute difference between the empirical and model-based correlation matrix is 0.03, and the maximum absolute difference between the empirical and model-based correlation matrix is 0.21 (with two discrepancies with an absolute difference >0.10), so the 1-factor structure is reasonable as a first-order approximation when considering that a 10×10 matrix with 45 correlations is approximated by a simple correlation matrix with 10 parameters. With a larger dimension (more stocks in the same sector), a 1-factor model with some weak conditional dependence (see [12]) could be a better dependence model.

Two parametric copula models are fitted to account for non-Gaussian dependence—1-factor with d=10 linking copulas that are all BB1, or all reflected BB1 (abbreviated as BB1r). These are referred to briefly as 1-factor BB1 and 1-factor BB1r. The details of these models are summarized in Appendix A.1 and Appendix A.2 in Appendix A; in particular, Appendix A.1 has the definition of the 2-parameter bivariate BB1 copula and some of its dependence properties, and Appendix A.2 has the definition of the 1-factor copula for *d* variables based on conditional independence of observed variables given a latent variable.

Table 1 has empirical and model-based lower and upper tail dependence measures: ${\hat{\lambda }}_{jk,L},\lambda_{jk,L}\left(\hat{\vartheta }\right)$, ${\hat{\lambda }}_{jk,U},\lambda_{jk,U}\left(\hat{\vartheta }\right)$. Model-based values are based on maximum likelihood estimates (MLEs) with 1-factor BB1r and 1-factor BB1. Table 2 has an empirical Spearman rank correlation as a central measure of dependence: ${\hat{\rho }}_{jk,S}$. The values of $\rho_{jk,S}\left(\hat{\vartheta }\right)$ for the two 1-factor copula models are quite close to the empirical values compared with some discrepancies for tail dependence measures. Table 3 has summaries, averaged over d(d−1)/10=45 bivariate margins. Table 1 and Table 3 show that tail inferences from different models with lower and upper tail dependence can be quite different, but the models can have similar inferences for central quantities.

The tail asymmetry of financial returns, with commonly more dependence in the joint lower tail than in the joint upper tail, is explained and discussed in [13,14]. The 1-factor BB1r model has a smaller AIC value than 1-factor BB1, and it better matches the empirical property of lower tail dependence, being often larger than upper tail dependence. However, the 1-factor BB1r model tends to overestimate the difference in the lower and upper tail dependence, and the 1-factor BB1 model tends to underestimate the difference in lower and upper tail dependence. This motivates the tilted likelihood in Section 3 with an appropriate “prior” so that model-based tail dependence measures are closer to empirical counterparts.

It has been observed in many data examples (see [15] and Chapter 7 of [9]) that model-based assessment of tail dependence may not be accurate. The more recent development of tail-weighted dependence measures in [16] allows for better assessment on the reliability of a parametric copula model for tail inferences, by comparing empirical and model-based directional tail-weighted measures.

## 3. Tilted Likelihood for 1-Factor Copula Model with Tail Dependence

This section has a modified log-likelihood using a prior based on previous data for tail dependence parameters in a 1-factor copula model (as given in Appendix A.2). The starting point is the copula-based log-likelihood after univariate margins have been estimated.

Assume that satisfactory univariate models ${\hat{F}}_{j}$ (1≤j≤d) have been fit to the random sample $\left\{\left(y_{i1},\ldots ,y_{id}\right):i=1,\ldots ,n\right\}$ and then transform to the uniform scale to $\left\{u_{i}\;=\;\left(u_{i1},\ldots ,u_{id}\right):i=1,\ldots ,n\right\}$ with $u_{ij}={\hat{F}}_{j}\left(y_{ij}\right)$ in the interval (0,1).

We consider mainly inference on dependence parameters for the data in the transformed uniform scale, considered as a realization of a random sample $\left\{U_{i}\right\}$ for a copula cumulative distribution function (cdf) $C_{U}\left(\cdot ;\vartheta \right)$, where $\vartheta =(\vartheta_{1},\ldots ,\vartheta_{d})$. The log-likelihood for a random sample of size *n* is:

(1) $$L\left(\vartheta \right)=L\left(\vartheta_{j}:j=1,\ldots ,d\right)=\sum_{i=1}^{n}\log c_{U}\left(u_{i};\vartheta_{1},\ldots ,\vartheta_{d}\right).$$

For the 1-factor copula based on BB1r (and other) linking copulas, there are lower bounds on components of the 2-dimensional $\vartheta_{j}$.

For likelihood inference, there is invariance to 1-1 transforms of $\vartheta_{j}$ to $\eta_{j}$, with the latter being functions of lower and upper tail dependence parameters. Specifically, $\eta_{j}=\left(\eta_{1j},\eta_{2j}\right)=(\log\left[\lambda_{jL}/\left(1-\lambda_{jL}\right],\log\left[\lambda_{jU}/\left(1-\lambda_{jU}\right)\right]\right)$ with $\lambda_{jL},\lambda_{jU}$ being the lower and upper tail dependence parameters for the bivariate copula linking variable *j* to the latent variable. Note that $\eta_{j}$ is unbounded. The tilted log-likelihood or log “posterior” is:

(2) $$\tilde{L}\left(\eta_{1},\ldots ,\eta_{d}\right)=\sum_{i=1}^{n}\log c_{U}\left(u_{i};\eta_{1},\ldots ,\eta_{d}\right)+\sum_{j=1}^{d}\log f_{H}\left(\eta_{j}\right)$$

where the density $f_{H}$ does not depend on *j*. The above is called the tilted log-likelihood because the goal is to obtain parameter estimates that put less weight in the middle of the data space and more weight in the tails based on “prior” expected behavior of how the lower and upper tail dependence parameters are related.

With the appropriate transformation, the prior can be taken as multivariate normal. For bivariate BB1r or BB1, $\eta_{j}$ is 2-dimensional, and $f_{H}$ is assumed to be bivariate normal. The latter is reasonable if the form of $\eta_{j}$ is chosen so that (2) is closer to a quadratic in a neighborhood of its mode. Asymptotic likelihood theory (see [17]) implies that the log-likelihood is quadratic in a neighborhood of the mode, as n→∞, but the adequacy of the approximation for moderate sample size *n* depends on the transform.

The justification of “independent” prior densities for different variables is based on some empirical checks for 1-factor copula construction with different bivariate linking copulas (with or without tail dependence). The inverse Hessian (roughly the covariance matrix of the sampling distribution of the MLE) of the negative log-likelihood in (1) for the 1-factor copula is close to the block diagonal, with a block for each $\eta_{j}$. The product form of the “prior” is based on an assumption of a “super-population” for the variables linked to the latent variable (e.g., stocks in a market sector). The density $f_{H}$ can be considered a frequency density of $\eta_{j}$ values over a large “super-population”.

A method is described in Section 4 to decide on choices for $f_{H}$.

Similar ideas to the tilted log-likelihood have been used to obtain an adjusted log-likelihood that corrects some undesirable behavior of the MLE, given in [18,19]. There are also some connections with variational Bayes inference such as when the posterior density is assumed to be approximated by a multivariate Gaussian density after a suitable transform so that parameters are unconstrained. However, with copula applications [20,21], parsimonious and possibly unrealistic assumptions are made for the covariance matrix (such as diagonal or factor structure) of the Gaussian density. The optimization involves a Kullback–Leibler divergence of the Gaussian approximation and the posterior. This differs from optimizing (2) with no constraints on the form of the Hessian matrix at the mode.

### Numerical Optimization for Posterior Mode and Hessian at Mode

The tilted log-likelihood has the penalized log-likelihood as an analogy so that standard numerical optimization methods can be used for estimating the mode and its Hessian.

The tilted log-likelihood in (2) and its log-likelihood counterpart in (1) are functions of 2d parameters for the 1-factor BB1r copula with *d* variables. For the log-likelihood, Ref. [22] discusses an efficient numerical procedure where the log-likelihood, gradient, and Hessian are analytically derived and coded in Fortran90, and all integrals are evaluated via Gauss–Legendre quadrature (see [23]).

The code is modified to handle the transform from BB1 parameters $\left(\theta_{j},\delta_{j}\right)$ to $\left(\eta_{1j},\eta_{2j}\right)$, and this requires care in using the chain rule for partial derivatives. The code for (2) and its gradient and Hessian are inputted into an efficient modified Newton–Raphson algorithm, as summarized in Section 6.2 of [9]. This leads to much faster computations than coding the negative of (2) in R and using a quasi-Newton method for numerical minimization based on numerical gradients and Hessians because many more iterations are needed compared with the modified Newton–Raphson. With the use of Fortran90 (for loops), analytic derivatives, and modified Newton–Raphson iterations, the time to deduce the posterior mode is decreased by a factor larger than 20 for 2d=40 parameters. Without the increased speed, the simulation study reported in Section 6 would take too much time. Also numerical optimization with the quasi-Newton method performs much worse as the number of parameters increases beyond 40.

With the negative Hessian at the mode of the tilted log-likelihood, the inverse Hessian can be used to obtain interval estimates for functions of the parameters.

## 4. Closer Match of Empirical and Model-Based Tail Dependence

Suppose diagnostic plots suggest tail dependence for all pairs of variables. Maximum likelihood estimation with a parametric copula might not provide good model-based estimates of tail dependence parameters or reliable inferences for tail-based quantities. In this section, a least squares method is used to obtain parameter estimates for the 1-factor copula that will make the empirical and model-based tail dependence parameters closer to each other. That is, there is an objective function to find copula parameters to better match model-based and empirical tail dependence parameters.

Let θ be the vector of all parameters $\left(\vartheta_{1},\ldots ,\vartheta_{d}\right)$. The *j*th component is $\vartheta_{j}=\left(\theta_{j},\delta_{j}\right)$ for the 1-factor BB1r or BB1 copula; see Appendix A.1 for the parametric BB1 family. The steps below assume that the 1-factor BB1r has lower AIC than 1-factor BB1 (empirical evidence from many applications of 1-factor copulas to GARCH-filtered stock returns).

1. Minimize negative of log-likelihood L(ϑ) in (1) to get MLE $\hat{\vartheta }$.
2. Get empirical matrix of lower tail dependence ${\hat{\lambda }}_{jk,L}$, upper tail dependence ${\hat{\lambda }}_{jk,U}$, central dependence Spearman rho ${\hat{\rho }}_{jk,S}$.
3. Minimize
  (3) $$\begin{array}{ccc} S(\vartheta ) & = & \frac{1}{2d(d-1)}\{\sum_{j<k}{\left[{\hat{\lambda }}_{jk,L}-\lambda_{jk,L}\left(\vartheta_{j},\vartheta_{k}\right)\right]}^{2}+\sum_{j<k}{\left[{\hat{\lambda }}_{jk,U}-\lambda_{jk,U}\left(\vartheta_{j},\vartheta_{k}\right)\right]}^{2}\\ & & +\sum_{j\neq k}{\left[{\hat{\rho }}_{jk,S}-\rho_{jk,S}\left(\vartheta_{j},\vartheta_{k}\right)\right]}^{2}\} \end{array}$$
  with $\hat{\vartheta }$ as starting point. Let the result be $\tilde{\vartheta }$.
4. Convert ${\tilde{\vartheta }}_{j}$ to ${\tilde{\lambda }}_{jV,L}=\lambda_{jV,L}\left({\tilde{\vartheta }}_{j}\right)$, ${\tilde{\lambda }}_{jV,U}=\lambda_{jV,U}\left({\tilde{\vartheta }}_{j}\right)$ as defined in (A6), using the BB1r linking copula for variable *j* and the latent variable *V*.
5. Transform to values in (−∞,∞): $\log[{\tilde{\lambda }}_{jV,L}/\left(1-{\tilde{\lambda }}_{jV,L}\right)]$ and $\log[{\tilde{\lambda }}_{jV,U}/\left(1-{\tilde{\lambda }}_{jV,U}\right)]$ for j=1,…,d.
6. Get the sample mean vector and covariance matrix for a sample of size *d* for the two transformed $\tilde{\lambda }$’s. The mean vector and covariance matrix are used as parameters for the bivariate normal prior $f_{H}$ in (2). For the tilted likelihood, use the parametrization
  $$\eta_{j}=\left(\log\left[\lambda_{jV,L}/\left(1-\lambda_{jV,L}\right)\right],\log\left[\lambda_{jV,U}/\left(1-\lambda_{jV,U}\right)\right]\right)$$
  for j=1,…,d.

The data set mentioned in Section 2 as used in [10] has 64 stocks in the finance sector, 21 stocks in the energy sector and 60 stocks in the health sector of S&P 500 (years 2013–2015). The above procedure is applied to 20 random stocks from the finance sector, 10 random stocks from the energy sector, and 20 random stocks from the health sector. Below in (4) to (6) are the mean vector and covariance matrix for $f_{H}$ for three cases:

(4) $$\begin{array}{ccc} \mu_{1} & = & \left(\begin{array}{c} 0.20\\ -0.38 \end{array}\right),\;\Sigma_{1}=\left(\begin{array}{cc} 0.163 & 0.134\\ 0.134 & 0.231 \end{array}\right),\;\rho_{1}=0.691; \end{array}$$

(5) $$\begin{array}{ccc} \mu_{2} & = & \left(\begin{array}{c} 0.04\\ -0.74 \end{array}\right),\;\Sigma_{2}=\left(\begin{array}{cc} 0.277 & 0.390\\ 0.390 & 0.830 \end{array}\right),\;\rho_{2}=0.813; \end{array}$$

(6) $$\begin{array}{ccc} \mu_{3} & = & \left(\begin{array}{c} -0.30\\ -1.05 \end{array}\right),\;\Sigma_{3}=\left(\begin{array}{cc} 0.121 & 0.140\\ 0.140 & 0.365 \end{array}\right),\;\rho_{3}=0.666. \end{array}$$

They are used as the parameters of three bivariate normal distributions. The three cases are used in subsequent sections to allow a sensitivity analysis of the parameters in $f_{H}$.

All three cases in (4) to (6) indicate stronger tail dependence in the joint lower tail compared with the joint upper tail because of the larger value in the first component of μ. Of the three cases, the first example has the strongest expected lower tail dependence because of largest first component of μ. For the first two cases, the median lower tail dependence is larger than 0.5 because of the positive value. The median upper tail dependence is less than 0.5 for all three cases.

## 5. Data Example with Prior and Tilted Likelihood

This section summarizes the application of the tilted log-likelihood for GARCH-filtered stock returns. Initially, three 1-factor copula constructions, with BB1, BB1r and BB7 bivariate linking copulas to the latent variable, were fitted with maximum likelihood for different subsets of stocks. Here, as is common from many empirical applications, the 1-factor copula based on BB1r is best, based on the AIC.

The tilted log-likelihood in (2) was then used for analysis of random subsets of stocks from the finance, energy and health sectors; these were different subsets from those used to determine the prior parameters (4)–(6). The qualitative conclusions are similar for different random subsets, so below we report details for one case of 20 randomly chosen finance stocks, considered one representative application of the theory in the preceding sections.

The numerical details below are based on 20 stocks with the tickers LNC, PGR, MMC, C, KEY, CBOE, BK, BEN, AXP, ALL, BAC, RF, AFL, ZION, DFS, CMA, MCO, GL, TRV, and BRK-B, used to determine the prior $f_{H}$, and 20 other stocks with the tickers L, CME, MTB, MKTX, AIZ, MET, SCHW, FITB, STT, HBAN, PFG, BLK, SPGI, CB, COF, TFC, WRB, JPM, FRCB, and ICE for applying the procedure in Section 3 and Section 4.

Inferences for tail dependence are compared for five cases below with summaries in Table 4.

1. 1-factor BB1r, $f_{H}$ based on finance sector stocks.
2. 1-factor BB1r, $f_{H}$ based on energy sector stocks.
3. 1-factor BB1r, $f_{H}$ based on health sector stocks.
4. 1-factor BB1, $f_{H}$ based on finance sector stocks.
5. 1-factor BB7, $f_{H}$ based on finance sector stocks.

The “parameters” of $f_{H}$ are $\left(\log\lambda_{L}/\left(1-\lambda_{L}\right),\;\log\lambda_{U}/\left(1-\lambda_{U}\right)\right)$ and have different transformations to the parameters (θ,δ) of BB1r, BBB1, BB7.

Table 4 shows that for BB1r, there is little sensitivity to the three priors (4)–(6). However, the worse fitting 1-factor BB1 and 1-factor BB7 models (based on last column of Table 4) do not lead to better matching with empirical dependence measures using the prior in (4). Overall, these latter two models fit worse in the middle of the data space, leading to smaller values for (2) at the mode.

For 1-factor BB1 with the tilted log-likelihood (2), we looked at the negative inverse Hessian (covariance matrix of normal approximation) in posterior mode for row 2 of Table 4. There is almost zero correlation of the parameters for different variable indices *j* (for different stocks). The inverse Hessian is too large to show in its entirety, but an extract of some entries is converted into standard deviations and correlations in Table 5 and Table 6.

### Bayesian Computing with STAN

Results based on the prior in (2) were also obtained via Bayesian computing with STAN (Hamiltonian Monte Carlo). Estimation for a 1-factor copula model via the Hamiltonian Monte Carlo is shown in [24], but their inferences do not include asymmetric tail dependence.

In Bayesian inference, the parameter vector $\Theta^{*}$ consists of both the (transformed) copula dependence parameters $\eta =(\eta_{1},\eta_{2},\ldots ,\eta_{d})$ and the latent variables $v=(v_{1},v_{2},\ldots ,v_{n})$ in (A3). We assume a joint independent uniform prior distribution for the latent variables and a (product of) bivariate normal prior for the copula dependence parameters for the bivariate linking copulas. The prior density is given by

$$\pi_{\Theta^{*}}\left(\theta^{*}\right)=\pi_{V}\left(v\right)\;\pi_{H_{1},\ldots ,H_{d}}\left(\eta \right)=\prod_{i=1}^{n}I\left(-1<v_{i}<1\right)\prod_{j=1}^{d}f_{H}\left(\eta_{j}\right),$$

where the mean and variance of the bivariate normal prior $f_{H}$ are given in (4)–(6). The “complete” likelihood function with the latent variables as parameters is

(7) $$\begin{array}{c} p_{U|\Theta^{*}}\left(u_{1},\ldots ,u_{n}|\theta^{*}\right)=\prod_{i=1}^{n}p_{U_{i}|V_{i},H_{1},\ldots ,H_{d}}\left(u_{i}|v_{i},\eta_{1:d}\right)=\prod_{i=1}^{n}\prod_{j=1}^{d}c_{jV}\left(u_{j},v_{i};\vartheta_{j}\left(\eta_{j}\right)\right), \end{array}$$

where $c_{jV}$ is given in Appendix A.2. Since the Bayesian estimation treats the latent variables as additional parameters, the likelihood function consists of the conditional density function given the latent variables instead of the joint density function. The posterior density function of the parameters (up to a constant) is

$$\pi_{\Theta |U}\left(\theta^{*}|u_{1},\ldots ,u_{n}\right)\propto p_{U,\Theta^{*}}\left(u,\theta^{*}\right)=p_{U|\Theta^{*}}u_{1},\ldots ,u_{n}|\theta^{*})\;\pi_{\Theta^{*}}\left(\theta^{*}\right).$$

To perform Bayesian inference on the (transformed) copula dependence parameters of the 1-factor model, we use the No-U-Turn sampler (NUTS) proposed by [25]. NUTs is an extension of the Hamiltonian Monte Carlo algorithm, implemented within the STAN framework developed by [26]. The 1-factor copula models with BB1 and reflected BB1 copulas are fitted to the GARCH-filtered returns in STAN. For the data example with results summarized in Table 5 and Table 6, the posterior statistics of η (including posterior means, standard deviations, and correlation matrix) are similar to the results obtained from maximizing the tilted likelihood function in (2). In comparison with Table 5, the median and maximum absolute differences are, respectively, (a) 0.006 and 0.033 for $\mu_{\eta }$’s, (b) 0.002 and 0.014 for $\sigma_{\eta }$’s, and (c) 0.023 and 0.059 for ρ’s.

From (7), it is seen that the log posterior is (up to a constant) equal to:

$$\tilde{L}\left(\eta_{1},\ldots ,\eta_{d},v\right)=\sum_{i=1}^{n}\sum_{j=1}^{d}\log c_{jV}\left(u_{j},v_{i};\vartheta_{j}\left(\eta_{j}\right)\right)+\sum_{j=1}^{d}\log f_{H}\left(\eta_{j}\right);$$

this is equivalent to the tilted log-likelihood in (2) after marginalizing over the latent variables v. Therefore, the two approaches should yield essentially the same result.

With a flat prior on the $\eta_{j}$, the posterior estimates should align with the maximum likelihood estimates. However, in the case of estimating BB1 or reflected BB1 copulas, identifiability issues arise when using a flat prior. The two parameters of BB1 or reflected BB1 are negatively dependent, which can result in different combinations of parameter values producing similar likelihood values. This issue might be overlooked in maximum likelihood estimation since it converges to one of the maxima, with an appropriate starting point for numerical optimization. However, it becomes evident in Bayesian estimation, where the model struggles to distinguish between different parameter values in the posterior distributions. We found that incorporating informative priors can effectively mitigate this problem. These priors leverage tail dependence measures to provide additional information about the relationship between the parameters, thereby improving the model’s ability to identify meaningful and interpretable parameter values.

## 6. Simulation Summary

This section has some simulation results for comparisons. Simulated data sets of size n=754 and d=20 are obtained to match the data example in Section 5; the algorithm for the simulation is in Algorithm 22 of [9]. For each simulated data set, $\left(\eta_{1j},\eta_{2j}\right)$ for j=1,…,d are generated at random from (4) and then a random sample $\left\{\left(u_{i1},\ldots ,u_{id}\right):i=1,\ldots ,n\right\}$ is generated from 1-factor BB1r based on the tail dependence parameters. For each simulated data set, as a sensitivity analysis, the log posterior in (2) for all three choices of $f_{H}$ based on (4)–(6) is maximized to obtain the mode and the approximate covariance matrix of the posterior density; also the MLE based on (1) is obtained.

The MLEs of the $\eta_{1j},\eta_{2j}$ parameters are transformed to the estimated θ,δ parameters of BB1r. Similarly, three sets of posterior modes for $\eta_{1j},\eta_{2j}$ parameters are transformed to estimate the θ,δ parameters. Then, the following root mean squares (rms) are computed:

(8) $${rms}_{m}={\left[{\left(2d\right)}^{-1}\sum_{j=1}^{d}\left({\left({\hat{\theta }}_{j}^{\left(m\right)}-\theta_{j}\right)}^{2}+{\left({\hat{\delta }}_{j}^{\left(m\right)}-\delta_{j}\right)}^{2}\right)\right]}^{1/2}$$

for the four sets of estimators. The superscripts m=1,2,3 indicate the three priors, and the superscript m=0 indicates maximum likelihood. Over 100 simulated data sets, the rms summaries are given in Table 7.

As expected, all three priors lead to closer estimates to the (θ,δ) parameters used to generate the simulated data sets than the MLEs. The three sets of $\left\{\left({\hat{\theta }}_{j}^{\left(m\right)},{\hat{\delta }}_{j}^{\left(m\right)}\right)\right\}$ for m=1,2,3 are relatively much closer to each other than with the MLE. For all simulated data sets, the value of the tilted log-likelihood (2) at the posterior mode is largest for prior (4) and smallest for prior (6).

Another summary in Table 8 is the closeness to the empirical $\lambda_{jk,L}$ and $\lambda_{jk,U}$ over the d2 pairs:

(9) $$\Delta_{M,m}={\left[d\left(d-1\right)/2\right]}^{-1}\sum_{j<k}\left|a_{jk,M}^{\left(m\right)}-a_{jk,M}^{empirical}\right|$$

where m∈{0,1,2,3} as above, and M∈{L,U,C} for lower tail dependence, upper tail dependence, and central dependence, with dependence measures $a\in \{{\hat{\lambda }}_{jk,L},{\hat{\lambda }}_{jk,U},{\hat{\rho }}_{jk,S}\}$, respectively.

From Table 8, there is better matching with the tilted log-likelihood for upper tail dependence but no improvement for lower tail dependence. The Spearman values $\rho_{jk,S}$ are much closer for empirical versus model based parameters.

In the comparison of the simulation results to those for the stock return data in Section 5, the improvements in using (2) are less. This can be explained as follows. For finance stock return data with stocks from one sector, the 1-factor structure with lower and upper tail dependence is reasonable, and BB1r linking copulas can be considered good approximations, and there might also be weak conditional dependence of some stock returns conditioned on the latent variable. That is, the 1-factor BB1r copula model has some small degree of model misspecification, and this can explain why tilting to obtain model-based tail dependence parameters to match empirical counterparts should lead to better tail inference.

## 7. Discussion

A method has been proposed for improved tail inference when preliminary data and likelihood analysis suggest asymmetric tail dependence. The approach of the tilted log-likelihood introduces a prior distribution involving lower and upper tail dependence parameters. Incorporating the prior places more weight on the behavior of the joint lower and upper tails compared with the center of the probability space, thereby improving the extreme value inference. This can account for a small degree of model misspecification in the parametric model. The prior is chosen so that model-based lower and upper tail dependence parameters can be a closer match to empirical counterparts for a previous data set that has some similar features to the data set under consideration.

For simpler exposition, the theory is applied to a 1-factor copula model that can handle non-Gaussian dependence structures with asymmetric tail dependence. The tilted log-likelihood approach can be extended to other structured factor copula models (e.g., bi-factor and1-factor with weak residual dependence) with asymmetric tail dependence, where a super-population assumption is reasonable for how observed variables are linked to latent variables.

Also, the approach can be applied to vine copula models with bivariate tail dependence for all pairs of variables by choosing bivariate copulas with lower and upper tail dependence in tree 1 of the vine. From [5], lower and upper upper tail dependence in the first vine tree lead to this property for all pairs of variables. By including a prior based on pairs of variables with stronger dependence and asymmetric tail dependence, there could be a better match of vine copula model-based tail dependence measures and empirical counterparts.

The skew-t copula (see [27]) can also be used for asymmetric tail dependence. However, the functional relation of copula parameters and tail dependence parameters is much more complicated than with the BB1 copula (latter in Appendix A.1) such that the tilted log-likelihood approach would be have to implemented with a different transform of the copula parameters.

Bayesian computing methods can be used if there are latent variables. Alternatively, a tilted log-likelihood similar to (2) can be optimized via (a) a quasi-Newton method if the total number of parameters is not large (say, fewer than 40), (b) a modified Newton–Raphson method if the analytical gradient and Hessian can be obtained, or (c) sequential estimation of parameters if possible (Section 5.5 of [9]). For methods (b) and (c), numerical optimization of the tilted log-likelihood is used to obtain the (approximate) posterior mode, and then the Hessian in this mode, in order to obtain interval estimates of the functions of parameters.

## Figures and Tables

**Table 1:** Matrices of tail dependence measures for 10 stock GARCH-filtered returns: model-based 1-factor BB1r, empirical, model-based 1-factor BB1, respectively. Lower (upper) tail dependence below (above) diagonal. Bootstrap standard errors (SEs) for estimates of lower and upper tail dependence are mostly in the range 0.04 to 0.075.

**Model-Based Tail-Dependence Based on MLE with 1-Factor BB1r**									
1.000	0.258	0.193	0.017	0.176	0.093	0.135	0.037	0.039	0.233
0.400	1.000	0.267	0.022	0.242	0.126	0.184	0.049	0.051	0.326
0.449	0.470	1.000	0.018	0.181	0.096	0.139	0.038	0.040	0.240
0.257	0.268	0.298	1.000	0.016	0.009	0.013	0.004	0.004	0.021
0.353	0.369	0.413	0.239	1.000	0.088	0.127	0.035	0.037	0.218
0.449	0.471	0.533	0.299	0.414	1.000	0.069	0.020	0.020	0.114
0.334	0.349	0.390	0.227	0.310	0.391	1.000	0.027	0.029	0.167
0.328	0.342	0.382	0.223	0.304	0.383	0.288	1.000	0.008	0.045
0.383	0.401	0.450	0.258	0.354	0.451	0.335	0.329	1.000	0.047
0.434	0.455	0.513	0.289	0.400	0.514	0.378	0.370	0.435	1.000
**Empirical Tail-Dependence Measures**									
1.000	0.418	0.259	0.087	0.196	0.233	0.200	0.160	0.144	0.239
0.397	1.000	0.238	0.141	0.275	0.285	0.247	0.144	0.163	0.333
0.362	0.382	1.000	0.118	0.268	0.326	0.275	0.108	0.202	0.347
0.184	0.168	0.217	1.000	0.130	0.142	0.080	0.189	0.157	0.079
0.283	0.310	0.344	0.231	1.000	0.178	0.209	0.111	0.132	0.290
0.281	0.364	0.494	0.132	0.307	1.000	0.182	0.078	0.161	0.339
0.235	0.274	0.375	0.207	0.320	0.301	1.000	0.060	0.131	0.308
0.267	0.275	0.219	0.234	0.207	0.273	0.201	1.000	0.137	0.109
0.246	0.312	0.373	0.156	0.262	0.333	0.151	0.279	1.000	0.198
0.284	0.293	0.450	0.224	0.301	0.438	0.255	0.284	0.276	1.000
**Model-Based Tail-Dependence Based on MLE with 1-Factor BB1**									
1.000	0.385	0.363	0.172	0.317	0.335	0.279	0.223	0.273	0.385
0.308	1.000	0.416	0.195	0.363	0.383	0.317	0.253	0.311	0.442
0.394	0.397	1.000	0.184	0.342	0.361	0.300	0.240	0.294	0.416
0.169	0.170	0.211	1.000	0.163	0.172	0.144	0.117	0.142	0.194
0.263	0.265	0.336	0.147	1.000	0.316	0.264	0.211	0.258	0.362
0.396	0.399	0.525	0.212	0.337	1.000	0.278	0.223	0.272	0.383
0.260	0.262	0.332	0.146	0.225	0.333	1.000	0.187	0.228	0.317
0.263	0.266	0.336	0.147	0.228	0.338	0.225	1.000	0.183	0.253
0.317	0.320	0.410	0.174	0.273	0.412	0.270	0.273	1.000	0.311
0.358	0.362	0.469	0.194	0.307	0.471	0.303	0.307	0.373	1.000

**Table 2:** Empirical Spearman rank correlation matrix for 10 GARCH-filtered stock returns. Bootstrap SEs for Spearman are in the range 0.017 to 0.036. Model-based Spearman rhos based on 1-factor BB1r and 1-factor BB1 are quite close to the respective empirical values.

Empirical Spearman Rho Central Dependence Measures									
1.000	0.745	0.597	0.321	0.534	0.584	0.439	0.444	0.567	0.565
0.745	1.000	0.633	0.376	0.605	0.620	0.491	0.519	0.577	0.639
0.597	0.633	1.000	0.323	0.551	0.709	0.581	0.506	0.561	0.723
0.321	0.376	0.323	1.000	0.347	0.378	0.352	0.508	0.362	0.388
0.534	0.605	0.551	0.347	1.000	0.569	0.494	0.390	0.501	0.558
0.584	0.620	0.709	0.378	0.569	1.000	0.557	0.498	0.558	0.720
0.439	0.491	0.581	0.352	0.494	0.557	1.000	0.325	0.420	0.599
0.444	0.519	0.506	0.508	0.390	0.498	0.325	1.000	0.467	0.485
0.567	0.577	0.561	0.362	0.501	0.558	0.420	0.467	1.000	0.567
0.565	0.639	0.723	0.388	0.558	0.720	0.599	0.485	0.567	1.000

**Table 3:** Summaries to indicate how well model-based tail dependence and central dependence approximate respective empirical values. The averages and fractions are over 102=45 bivariate margins.

Summary	Value
average $|{\hat{\lambda }}_{jk,L}-\lambda_{jk,L}\left(\hat{\vartheta }\right)|$ for 1-factor BB1r	0.088
average $|{\hat{\lambda }}_{jk,U}-\lambda_{jk,U}\left(\hat{\vartheta }\right)|$ for 1-factor BB1r	0.101
average $|{\hat{\rho }}_{jk,S}-\rho_{jk,S}\left(\hat{\vartheta }\right)|$ for 1-factor BB1r	0.035
average $|{\hat{\lambda }}_{jk,L}-\lambda_{jk,L}\left(\hat{\vartheta }\right)|$ for 1-factor BB1	0.043
average $|{\hat{\lambda }}_{jk,U}-\lambda_{jk,U}\left(\hat{\vartheta }\right)|$ for 1-factor BB1	0.088
average ${\hat{\rho }}_{jk,S}-\rho_{jk,S}\left(\hat{\vartheta }\right)$ for 1-factor BB1	0.036
average $\left({\hat{\lambda }}_{jk,L}-{\hat{\lambda }}_{jk,U}\right)$	0.088
average $\left(\lambda_{jk,L}\left(\hat{\vartheta }\right)-\lambda_{jk,U}\left(\hat{\vartheta }\right)\right)$ for 1-factor BB1r	0.275
average $\left(\lambda_{jk,L}\left(\hat{\vartheta }\right)-\lambda_{jk,U}\left(\hat{\vartheta }\right)\right)$ for 1-factor BB1	0.021
fraction ${\hat{\lambda }}_{jk,L}>{\hat{\lambda }}_{jk,U}$	40/45
fraction $\lambda_{jk,L}\left(\hat{\vartheta }\right)>\lambda_{jk,U}\left(\hat{\vartheta }\right)$ for 1-factor BB1r	45/45
fraction $\lambda_{jk,L}\left(\hat{\vartheta }\right)>\lambda_{jk,U}\left(\hat{\vartheta }\right)$ for 1-factor BB1	29/45

**Table 4:** Closeness to corresponding empirical values to model-based ML or posterior modal $\eta_{L}.\eta_{U}$ values for lower tail dependence $\lambda_{jk,L}$, upper tail dependence $\lambda_{jk,U}$ and central dependence parameters $\rho_{jk,S}$ of d(d−1)/2 pairs; d = 10 GARCH-filter stock returns. The quantiles in columns 2 to 4 are from the average absolute difference over d(d−1)/2 pairs.

Case	$\lambda_{L}$	$\lambda_{U}$	$\rho_{S}$	Objective (2)
BB1r, MLE	0.094	0.116	0.034	–
BB1r, prior (4)	0.077	0.043	0.034	5766
BB1r, prior (5)	0.081	0.054	0.034	5766
BB1r, prior (6)	0.074	0.046	0.035	5743
BB1, prior (4)	0.078	0.078	0.036	5737
BB7, prior (4)	0.140	0.134	0.062	5549

**Table 5:** Posterior mode and standard deviation (SD) of $\eta_{1j},\eta_{2j}$ parameters. Note that $\mu_{\eta_{1j}}>\mu_{\eta_{2j}}$ implies strong lower tail dependence than upper tail dependence for variable *j* with the latent variable. $\mu_{\eta_{1j}}>0$ means that the estimated lower tail dependence with latent variable exceeds 0.5. The SD σ values come from the square root of diagonal values of the negative inverse Hessian at the mode. The correlation values for each diagonal 2×2 block come from converting a covariance matrix to a correlation matrix.

Variable *j*	$\mu_{\eta_{1j}}$	$\mu_{\eta_{2j}}$	$\sigma_{\eta_{1j}}$	$\sigma_{\eta_{2j}}$	$\rho_{\eta_{1j},\eta_{2j}}$
1	0.292	−0.351	0.081	0.202	−0.398
2	−0.308	−0.797	0.101	0.217	−0.315
3	0.447	−0.010	0.081	0.186	−0.442
4	−0.771	−1.422	0.119	0.243	−0.163
5	0.250	−0.904	0.076	0.227	−0.274
6	0.758	−0.261	0.068	0.215	−0.373
7	0.435	−0.390	0.076	0.209	−0.379
8	0.560	−0.165	0.075	0.200	−0.411
9	0.553	−0.137	0.075	0.200	−0.422
10	0.657	−0.314	0.070	0.213	−0.372
11	0.803	−0.255	0.067	0.215	−0.366
12	0.460	−0.755	0.071	0.224	−0.292
13	−0.108	−1.312	0.086	0.234	−0.197
14	0.134	−0.859	0.081	0.226	−0.292
15	0.253	−0.637	0.079	0.219	−0.336
16	0.600	0.101	0.078	0.186	−0.450
17	−0.149	−1.240	0.089	0.233	−0.216
18	0.533	−0.036	0.078	0.192	−0.437
19	0.107	−0.734	0.083	0.221	−0.323
20	−0.393	−1.228	0.101	0.235	−0.215

**Table 6:** Part of negative inverse Hessian at posterior mode, that is, the posterior covariance for variables $\mu_{\eta_{1j}},\mu_{\eta_{2j}}$, j∈{1,7,13}. Note that near the block diagonal form, the matrix is diagonal 2×2 block dominant.

j=1		j=7		j=13	
0.00662	−0.00655	−0.00002	−0.00001	0.00005	−0.00003
−0.00655	0.04093	−0.00002	−0.00025	0.00001	0.00052
−0.00002	−0.00002	0.00570	−0.00600	0.00005	0.00000
−0.00001	−0.00025	−0.00600	0.04388	0.00002	0.00029
0.00005	0.00001	0.00005	0.00002	0.00737	−0.00396
−0.00003	0.00052	0.00000	0.00029	−0.00396	0.05473

**Table 7:** Values of average difference in values in (8) over 100 simulated data sets. Also included are the fraction of times that the posterior mode from (2) is closer to the “true” vector compared with the MLE, and lower/upper quartiles of (8).

*m*	Average ${\mathrm{rms}}_{0}-{\mathrm{rms}}_{m}$	Fraction ${\mathrm{rms}}_{0}>{\mathrm{rms}}_{m}$	Q1 rms*_m_*	Q3 rms*_m_*
0			0.085	0.101
1	0.023	0.95	0.066	0.079
2	0.019	0.92	0.071	0.082
3	0.013	0.80	0.074	0.091

**Table 8:** Values of average difference for values of (9) in 100 simulated data sets. Also, the fraction of times that the posterior mode from (2) improves on the MLE based on (9), and lower/upper quartiles of (9).

*m*	*M*	avg $\Delta_{M,0}-\Delta_{M,m}$	Fraction $\Delta_{M,m}<\Delta_{M,0}$	Q1 $\Delta_{M,m}$	Q3 $\Delta_{M,m}$
0	L			0.059	0.078
1	L	0.001	0.46	0.060	0.077
2	L	0.001	0.48	0.060	0.077
3	L	0.000	0.37	0.060	0.079
0	U			0.073	0.129
1	U	0.011	0.87	0.065	0.116
2	U	0.004	0.70	0.070	0.122
3	U	0.011	0.92	0.065	0.114

## Data Availability

The data presented in this study are available on request from the corresponding author.

## Appendix A. Background Results on Copulas and Dependence Concepts

The appendix consists of subsections of known results in order to make this article more self-contained. Details of individual topics are in [9] if there are no additional references.

### Appendix A.1. BB1 Copula Family and Tail Dependence Properties

If (U,V)∼C for a bivariate copula *C*, the reflected copula $\hat{C}\left(u,v\right)$ is the distribution of the reflection (1−U,1−V). Simple probability calculations lead to:

$$\hat{C}\left(u,v\right)=u+v-1+C\left(1-u,1-v\right),\;0\leq u,v\leq 1.$$

The survival function of *C* is related; it is defined as

(A1) $$\bar{C}\left(u,v\right)=\mathrm{Pr}\left(U>u,V>v\right)=1-u-v+C\left(u,v\right),\;0\leq u,v\leq 1,$$

so that $\hat{C}\left(u,v\right)=\bar{C}\left(1-u,1-v\right)$.

The bivariate 2-parameter BB1 copula family is useful to handle asymmetric lower and upper tail dependence. Asymmetric here refers to copulas where *C* is different from $\hat{C}$. For θ>0,δ>1,

(A2) $$C_{\mathrm{BB}1}\left(u,v;\theta ,\delta \right)={\left\{1+{\left[{\left(u^{-\theta }-1\right)}^{\delta }+{\left(v^{-\theta }-1\right)}^{\delta }\right]}^{1/\delta }\right\}}^{-1/\theta },\;0\leq u,v\leq 1.$$

The reflected copula is: $C_{\mathrm{BB}1r}\left(u,v;\theta ,\delta \right)=u+v-1+C_{\mathrm{BB}1}\left(1-u,1-v;\theta ,\delta \right)$.

The BB1 copula and its reflection are the most useful for handling asymmetric tail dependence. Other choices for this property are BB7 and skew-t (see [27]). The BB1 family has the nice property that there is increasing concordance or positive dependence as θ and δ increase with independence as the lower bound of the parameter space is reached and comonotonicity (perfect dependence) as one of θ or δ goes to infinity. The BB7 copula does not have the concordance property over the entire parameter space.

Tail dependence parameters (as functions of copula family) are $\lambda_{L}\left(C_{\mathrm{BB}1}\right)=2^{-1/(\delta \theta )}$ and $\lambda_{U}\left(C_{\mathrm{BB}1}\right)=2-2^{1/\delta }$ as functions of θ and δ. Then, $\lambda_{U}\left(C_{\mathrm{BB}1r}\right)=\lambda_{L}\left(C_{\mathrm{BB}1}\right)$ and $\lambda_{L}\left(C_{\mathrm{BB}1r}\right)=\lambda_{U}\left(C_{\mathrm{BB}1}\right)$ because the reflection reverses the joint upper and lower tails. The range of $\left(\lambda_{L},\lambda_{U}\right)$ is ${\left(0,1\right)}^{2}$. To go from tail dependence parameters to copula parameters, the transforms are:

$$\begin{array}{ccc} & & \delta =\left[\log2\right]/\log\left(2-\lambda_{U}\right),\;\theta =\log\left(2-\lambda_{U}\right)/\left(-\log\lambda_{L}\right)\;\mathrm{for}\mathrm{BB}1,\\ & & \delta =\left[\log2\right]/\log\left(2-\lambda_{L}\right),\;\theta =\log\left(2-\lambda_{L}\right)/\left(-\log\lambda_{U}\right)\;\mathrm{for}\mathrm{BB}1r,\\ & & \delta =\left[\log2\right]/\left[-\log-\lambda_{L}\right),\;\theta =\left[\log2\right]/\log\left(2-\lambda_{U}\right)\;\mathrm{for}\mathrm{BB}7. \end{array}$$

For the data example in Section 5, the BB7 1-factor copula is fitted much worse, but it is included to show that the “prior” in (2) is independent of the tail-dependent family used for linking copulas to the latent variable.

### Appendix A.2. 1-Factor Copula Construction

For the 1-factor copula model, it assumes conditional independence of $U=(U_{1},\ldots ,U_{d})$, given a latent variable V∼U(0,1). Let $C_{jV}\left(\cdot ,\vartheta_{j}\right)$ (for j=1,…,d) be the bivariate copula cdf for the *j*th variable with the latent variable *V*. The *d*-variate copula cdf and density for $U=(U_{1},\ldots ,U_{d})$ are:

(A3) $$\begin{array}{ccc} C_{U}\left(u;\vartheta_{1},\ldots ,\vartheta_{d}\right) & = & \int_{0}^{1}\prod_{j=1}^{d}C_{j|V}\left(u_{j}|v;\vartheta_{j}\right)\;dv, \end{array}$$

(A4) $$\begin{array}{ccc} c_{U}\left(u;\vartheta_{1},\ldots ,\vartheta_{d}\right) & = & \int_{0}^{1}\prod_{j=1}^{d}c_{jV}\left(u_{j},v;\vartheta_{j}\right)\;dv, \end{array}$$

where $C_{j|V}\left(u_{j}|v;\vartheta_{j}\right)=\partial C_{jV}\left(u_{j},v;\vartheta_{j}\right)/\partial v$ is the conditional distribution of $\left[U_{j}|V=v\right]$ and $c_{jV}\left(u_{j},v,\vartheta_{j}\right)=\partial^{2}C_{jV}\left(u_{j},v;\vartheta_{j}\right)/\partial v\partial u_{j}$ is the corresponding copula density. The (j,k) bivariate margin is

(A5) $$C_{jk}\left(u_{j},u_{k}\right)=\int_{0}^{1}C_{j|V}\left(u_{j}|v;\vartheta_{j}\right)C_{k|V}\left(u_{k}|v;\vartheta_{k}\right)\;dv.$$

The Spearman’s rho for $\left(U_{j},U_{k}\right)$ is $\mathrm{Cor}\;(U_{j},U_{k})$. It is numerically best to compute it via a 2-dimensional numerical integral

$$12\int_{0}^{1}\int_{0}^{1}C_{jk}\left(u_{u},u_{k}\right)\;du_{j}\;du_{k}-3$$

to avoid a possible unbounded copula density $c_{jk}$. For (A5), this becomes

$$12\int_{0}^{1}\left(\int_{0}^{1}C_{j|V}\left(u_{j}|v\right)\;du_{j}\right)\left(\int_{0}^{1}C_{j|V}\left(u_{k}|v\right)\;du_{k}\right)\;dv-3=:12\int_{0}^{1}a_{j}\left(v\right)\;a_{k}\left(v\right)\;dv-3;$$

This can be computed via a 1-dimensional Gauss–Legendre quadrature over $n_{q}$ quadrature points $v_{1},\ldots ,v_{n_{q}}$ for *v*, and $a_{j}\left(v\right)$ and $a_{k}\left(v\right)$ (with respect to $u_{j}$ and $u_{k}$) can be separately evaluated via the Gauss–Legendre quadrature for $v_{1},\ldots ,v_{n_{q}}$. See [23] for Gaussian quadrature theory.

Suppose $C_{jV}$ has lower and upper tail dependence for all *j*, such as with BB1 or reflected BB1 copulas. Define the survival function ${\bar{C}}_{jV}$ as in (A1). Then, from Section 2.18 of [9], there are functions $b_{jV}$ and $b_{jV}^{*}$ for lower and upper tails, respectively, such that for $w_{j}>0$ and w>0,

$$\begin{array}{ccc} C_{jV}\left(uw_{j},uw\right)/u & \to & b_{jV}\left(w_{j},w\right),\;u\to 0^{+},\\ {\bar{C}}_{jV}\left(1-uw_{j},1-uw\right)/u & \to & b_{jV}^{*}\left(w_{j},w\right),\;u\to 0^{+},\\ C_{j|V}\left(uw_{j}|uw\right) & \to & b_{j|V}\left(w_{j},w\right)=\partial b_{jV}\left(w_{j},w\right)/\partial w,\;u\to 0^{+},\\ {\bar{C}}_{j|V}\left(1-uw_{j}|1-uw\right) & \to & b_{j|V}^{*}\left(w_{j},w\right)=\partial b_{jV}^{*}\left(w_{j},w\right)/\partial w,\;u\to 0^{+}. \end{array}$$

Lower and upper tail dependence parameters are defined in Section 2. The conditions of Theorem 8.76 of [9] are satisfied with BB1 copulas, and it implies for variable *j* and *k* (j≠k) that the lower and upper table dependence parameters for $\left(U_{j},U_{k}\right)$ are

(A6) $$\lambda_{jk,L}=\int_{0}^{\infty }b_{j|V}\left(1|z\right)b_{k|V}\left(1|z\right)\;dz,\;\lambda_{jk,U}=\int_{0}^{\infty }b_{j|V}^{*}\left(1|z\right)b_{k|V}^{*}\left(1|z\right)\;dz.$$

For BB1,

$$\begin{array}{ccc} b_{jV}\left(w_{j},w\right) & = & {\left(w_{j}^{-\delta \theta }+w^{-\delta \theta }\right)}^{-1/(\delta \theta )},\\ b_{jV}^{*}\left(w_{j},w\right) & = & w_{1}+w-{\left(w_{j}^{\delta }+w^{\delta }\right)}^{1/\delta },\\ b_{j|V}\left(1|v\right) & = & {\left(1+v^{\delta \theta }\right)}^{-1-1/(\delta \theta )},\\ b_{j|V}^{*}\left(1|v\right) & = & 1-{\left(1+v^{-\delta }\right)}^{1/\delta -1}. \end{array}$$

For reflected BB1, the lower and upper quantities are reversed, as the reflection just changes the upper (lower) quadrant to the lower (upper) quadrant.

Reflected BB1 and BB1 copulas usually fit better than the BB7 copula for stock return data with factor or vine copulas. From the concordance property of the BB1 copula, there is more lower and upper tail dependence as either of the two parameters increase.

### Appendix A.3. Normal Scores and Use for Diagnostics

Let $y_{1},\ldots ,y_{n}$ be a sample of size *n* for a variable *y*. The rank transform to the standard normal is

$${\hat{z}}_{i}=\Phi^{-1}\left(\left(\mathrm{rank}\left(y_{i}\right)-0.5\right)/n\right),$$

where a larger *y* value attains a larger rank (rank 1 for smallest and rank *n* for largest (among the sorted values of $y_{1},\ldots ,y_{n}$). We refer to the $\left\{{\hat{z}}_{i}\right\}$ as the normal scores for *y*.

If there are *d* variables and a sample $\left\{\left(y_{i1},\ldots ,y_{id}\right):i=1,\ldots ,n\right\}$, then one can obtain vectors of normal scores $\left\{\left({\hat{z}}_{i1},\ldots ,{\hat{z}}_{id}\right):i=1,\ldots ,n\right\}$ with ranking performed separately for each variable. Pairs of variables have strong monotone dependence if the correlation of their normal score transforms is large. Figure 1 has examples of bivariate normal scores plots for pairs of GARCH-filtered returns. The plots are used to check for departures from elliptically shaped clouds of points, which are expected if the Gaussian copula fits well.

### Appendix A.4. GARCH Time Series

A summary of the copula-GARCH models is as follows. Let $P_{t}$ (t=0,1,…,n) be the price time series of a financial asset such as a market index or stock; the time index could be day, week, or month. The (log) return $R_{t}$ is defined as $\log(P_{t}/P_{t-1})$. For *d* assets, denote the returns as $R_{t1},\ldots ,R_{td}$ at time *t*. For each financial return variable, a common choice is the GARCH(1,1) time series filter, with innovation distribution being the symmetric (or asymmetric) Student t_*ν*_ distribution with variance 1 and ν>2; see Section 4.3.6 of [28].

Let $F_{j}\left(\cdot ,\nu_{j}\right)$ be the distribution of the innovation for the *j*th univariate marginal model:

(A7) $$R_{tj}=\mu_{j}+\sigma_{tj}Z_{tj},\;\sigma_{tj}^{2}=\omega_{j}+\alpha_{j}R_{t-1,j}^{2}+\beta_{j}\sigma_{t-1,j}^{2},\;j=1,\ldots ,d,\;t=1,\ldots ,n,$$

where for each *j*, $\omega_{j}>0$, $\alpha_{j}>0$, $\beta_{j}>0$, the $Z_{tj}$ are assumed to be innovations over *t*, and the random $\sigma_{tj}^{2}$ depends on $R_{t-1,j}^{2}$ and $\sigma_{t-1,j}^{2}$. For stationarity, $\alpha_{j}+\beta_{j}<1$ for all *j*. The vectors $\left(Z_{t1},\ldots ,Z_{td}\right)$ for t=1,…,n are assumed to be independent and identically distributed with distribution:

$$F_{Z}\left(z;\nu_{1},\ldots ,\nu_{d},\vartheta \right)=C\left(F_{1}\left(z_{1};\nu_{1}\right),\ldots ,F_{d}\left(z_{d};\nu_{d}\right);\vartheta \right),$$

where ϑ is a dependence parameter of a *d*-dimensional copula *C*. Sometimes, an autoregressive coefficient $\phi_{j}$ is also added when $\mu_{j}$ in (A7) is replaced by $\mu_{j}+\phi_{j}R_{t-1,j}$. The flexible choice of the copula families includes vine and factor copula constructions.
